# Consumers’ Perceptions and Preferences for Bitterness in Vegetable Foods: The Case of Extra-Virgin Olive Oil and Brassicaceae—A Narrative Review

**DOI:** 10.3390/nu11051164

**Published:** 2019-05-24

**Authors:** Carla Cavallo, Gianni Cicia, Teresa Del Giudice, Raffaele Sacchi, Riccardo Vecchio

**Affiliations:** Department of Agricultural Sciences, Università degli Studi di Napoli Federico II, 80055 Portici, Italy; cicia@unina.it (G.C.); agriqual@unina.it (T.D.G.); sacchi@unina.it (R.S.); riccardo.vecchio@unina.it (R.V.)

**Keywords:** bitter taste, consumers’ preferences, consumers’ perceptions, vegetable foods, extra virgin olive oil, *Brassicaceae*, sensory acceptability

## Abstract

The presence of some healthy phytochemicals in food can be paired with high bitterness, and consumers have a widespread avoidance toward bitter-tasting food. This causes a gap between preferences and healthy needs of consumers. Therefore, this review collected insights from literature belonging to different discipline domains in order to have a broad view of the current state-of-the-art about biochemical aspects and consumers’ perceptions and preferences toward foods with an enhanced bitter taste. In detail, we focused on two core products of the Mediterranean diet: Extra-virgin olive oil (EVOO) and Brassicaceae, both characterized by specific phytochemicals having strong healthy properties and bitter-pungent taste. Results suggested that, although bitter taste is a general driver of dislike, some exceptions can be represented by: niches of consumers (e.g., innovators and organic buyers), foods consumed with specific purposes (e.g., coffee, chocolate, and alcoholic beverages). The level of bitterness perceived by the consumers can be modulated through exposure, information on benefits, and elements within the environment (e.g., music). Thus, these insights can be used to develop specific campaigns aimed at promoting bitter (healthy) food, considering also the key role that could be played by food pairings.

## 1. Introduction

Extra-virgin olive oil (EVOO) and Brassica vegetables, such as broccoli and rocket salad, are at the core of the Mediterranean diet. The Mediterranean diet is recognized as one of the most beneficial forms of nutrition due to its large share of plant-based foods and the high monounsaturated content of liberally-consumed olive oil [1] However, the healthy power of vegetables can have several sources of variability, as healthy bioactive substances having antioxidant and other functional properties are secondary metabolites produced in edible plants for eco-physiological purposes and are mainly influenced by genetic, environmental, pedologic, agronomical, and technological factors [2]. These bioactive molecules very often give to the food a bitter taste [3]. This is the case of several vegetables characterized by specific bitter-pungent phytochemicals, like glucosinolates in Brassicaceae (or Cruciferae) family [4] and secoiridoids in extra-virgin olive oil (EVOO) [5]. In this review we will focus on these two vegetable product categories, not investigating the specificities of other bitter compounds, such as as peptides, present in other animal foods (e.g., meat and cheeses).

Although the intake of vegetable products is constantly paired with positive effects on health [6], detailed studies proved that this association is not always true. In fact, although there is some evidence about how cooking can enhance the bioavailability of some antioxidants [7,8], a cohort study on mortality found a modest association between a longer life and cooked vegetables intake, while a strong effect on a longer life was delineated for raw vegetables intake [9]. Analogously, being less bitter, vegetable foods have a diminished healthy power. In this scenario we can highlight a deviation: While consumers of developed countries show a high preference for healthy food [10], they do not prefer food with a bitter and pungent taste [11]. 

The reasons behind these preferences can largely be ascribed to evolution [12,13], as bitter taste signals intoxicating substances. In the case of edible vegetables, on the contrary, bitterness has no real intoxicating effect, but a beneficial association with health [14,15]. An example of such mechanism is represented by the preparation of Brassicaceae. This type of vegetable, when cooked, on the contrary to the behavior of carotenoid-rich vegetables, like carrots and tomatoes [16,17], hold a lower health association due to the depletion of beneficial phytochemicals induced by cooking [18], even if consumers generally prefer such vegetables cooked as they are less bitter and pungent [19]. 

Over the years, the breeding and the food industry took advantage of such a situation, increasing consumers’ acceptance of different foods, following two main strategies: The elimination of bitter substances from vegetables [20] and the use of sweet masking substances [21]. In either case, the outcome is that the foods available on the final markets tend to be significantly-less healthy and consumers, through a lack of exposure, are not accustomed to bitter taste and will further avoid bitter foods in future [22]. A review by Drewnowski and Gomez-Carneros [23] dealt extensively with those issues, discussing in detail the content of the main bitter phytonutrients in common plant foods, with reference to natural vegetable food sources and average content range. In particular, Authors described bitter flavonoids in citrus fruits, bitter phenols in tea, chocolate, and red wine, bitter isoflavones in soybeans, bitter glucosinolates in Brassica vegetables and their pungent isothiocyanate derivatives, and debittering practices applied by the food industry. In this framework, the current review aims to go beyond such research by updating and discussing the wealth of knowledge on consumer bitter perceptions and preferences, distributed in different disciplines domain, providing a basis for further investigations and research. The endorsed point of view of this work is the one focused on consumer’s preferences and the collected insights will be discussed in terms of future possibilities for consumer’s research. We hereafter refer to the term “preference” as the choice to buy a product based on consumers’ assessment of the utility gained from product selection [24]. While we will use the term “consumer perception” as the evaluative criteria applied by individuals to form beliefs and develop attitudes towards a product [25], and the term acceptability as the degree of consumer appreciation [26]. We also bear in mind that a vast amount of literature has established that quality perception can be profoundly influenced by extrinsic product aspects [27] and contextual and environmental factors [28].

We will discuss two main case studies: EVOO and Brassicaceae due to their healthy and sensory characteristics. These two products play a key role in the Mediterranean diet [29,30,31,32]. Mediterranean diet is known for its high content of antioxidants and anti-inflammatory compounds [32]. Most of these healthy substances are found in EVOO and Brassicaceae. Unfortunately, the content in antioxidant substances in these two products is closely related to the bitterness. While for EVOO hedonic liking and consumers’ preferences have been extensively dealt in previous studies, highlighting a discrepancy between European standards of quality and final users’ preferences [33,34,35]; whereas for Brassicaceae a very limited amount of scientific literature is available, underlining the importance of further investigations [36]. It is important to note that European Regulations for EVOO standardize also the sensory profile for Protected Designation of Origin (PDO) and Protected Geographical Indication (PGI) products. Among the sensory descriptors included in such requirements, the mandatory indication of the level of bitterness is also included.

The present paper is structured as follows. The collected insights, since originating from different disciplines domains, have been divided in three sections, according to the core investigated topic: (i) Psychological studies, mainly related to consumer quality perception; (ii) studies on biochemical/physiological aspects of bitter taste; and (iii) studies on single product perception and preferences; within this section two sub-sections were highlighted according to the articles that dealt specifically with EVOO and Brassicaceae. Then conclusions browse the main lessons learnt from the literature and new perspectives are presented.

### Physiology of Taste in Brief

From the physiological point of view, the perception of bitterness is due to activation of seven-transmembrane receptors (the T2Rs) and trigeminal nerve endings, located within the oral, sensitive to chemical stimuli [37]. The T2Rs, such as T2R38, coupled to the G protein gustducin are responsible for the human ability to taste bitter substances [38]. The T2R family in humans is thought to comprise about 25 different taste receptors, some of which can recognize a wide variety of bitter-tasting compounds [39].

**Figure 1:**
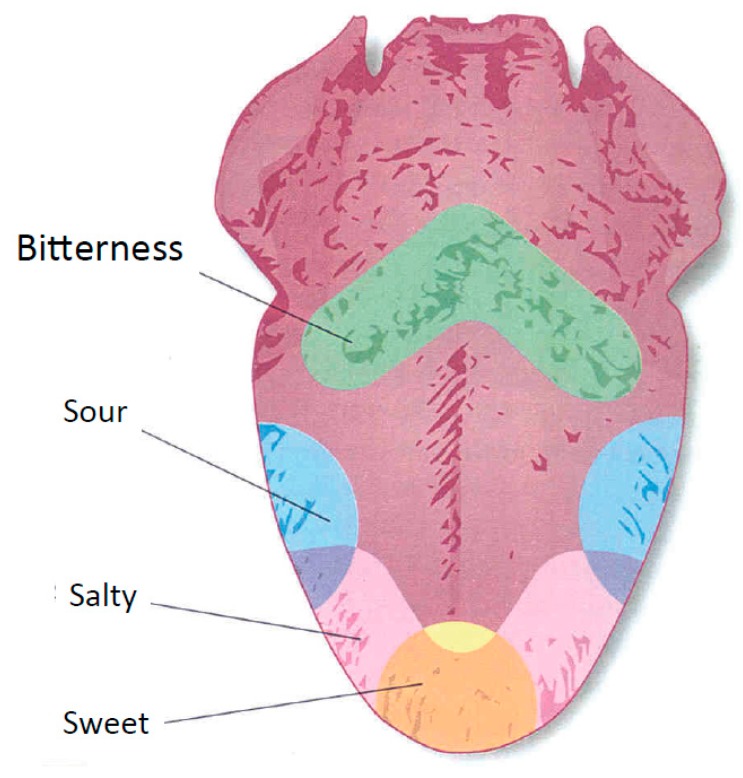
Schematic picture of the tongue with indication of the “V area” (green) in which bitterness is particularly perceived.

Bitter tastes are triggered by the binding of molecules to G protein-coupled receptors on the cell membranes of taste buds. Tongue and oral epithelium are covered by taste buds composed of about 50 modified epithelial cells, some of which are supporting cells called sustentacular cells and others of which are taste cells. A large number of taste buds surround the circumvallate papillae, which form a “V line” on the surface of the posterior tongue (Figure 1). For the bitter taste sensations, the portions of the receptor protein molecules that protrude through the apical membranes activate second-messenger transmitter substances inside the taste cells, and these second messengers cause intracellular chemical changes that elicit the taste signals [37].

Recent evidence indicates a genetic variation between populations in sensitivity to bitter taste, with a frequency of non-tasters that can vary from as low as 7% to 40% [40]. Surprisingly the so-called taste receptors were identified not only in the mouth but also in the gut, the pancreas, and the brain, thus suggesting that they may play a significant role in nutrient recognition and regulation [41,42,43]. 

On the other hand, liking for taste stimuli is generally strongly influenced by inborn (innate) factors [44]. Sweet foods are innately preferred by most or all herbivores and omnivores, presumably since sweetness reflects the presence of caloric sugars in plants. On the contrary, bitter taste signals the presence of potentially toxic compounds and, hence, substances that are bitter are generally disliked and avoided [45]. From an evolutionary perspective, a sensitive perception of bitter taste was strictly linked to survival during the Paleolithic period: The hunter-gather men that were able to perceive bitterness were also able to avoid dangerous foods. On the other hand, also several compounds with antimicrobial activity have a bitter taste (such as many spices), and for populations living in regions where malaria was endemic the insensitivity to this taste allowed survival. Thus, looking at the human history, the different grade of bitter perceptions seems strictly co-evolved with society habits and environment [46].

Food preferences always evolve during the lifespan. Generally, infants or children have more preferences than adults and these preferences can be modified by experience [47]. Thus, both individual experience and social environment highly influence food liking and, as consequence, dietary choice. Nevertheless, with experience even bitter vegetables can come to be liked although whether the bitterness of these is actually liked or whether the other flavor characteristics are sufficient to overcome the dislike of bitterness is not known as well [48,49].

Subsequently, astringency and pungency sensations are often associated to bitterness in the consumption of some vegetable foods. Astringency is defined by the American Society for Testing of Materials as “the complex of sensations due to shrinking, drawing, or puckering of the epithelium as a result of exposure to substances such as alums or tannins” [50].

The current model describing astringency is based on precipitation of salivary proline-rich proteins by tannins and polyphenols [51,52] and/or altered salivary lubrication [50]. Because dryness from astringency is detected by oral tissues this suggests other interactions, possibly through direct alteration of the lubricating mucosal pellicle, which may also expose the oral mucosa below. A loss of mucosal lubrication is likely to be fundamental in astringency development and it seems likely that astringent stimuli alter the salivary bulk, saliva rheology, and the saliva pellicle leading to an increase of friction in the oral cavity [50]. 

The role of receptors in astringency perception has ben also studied. Tea polyphenols, particularly those including the galloyl ring, have been shown to activate human bitter receptors hT2Rs [53]. Epigallocatechin gallate (EGCG) also activates transient receptor protein channel, resulting in a Ca^2+^ response, which could occur on the tongue and contribute to astringency in the mouth [54].

In the end, pungency (also spiciness or hotness) is a burning sensation induced by a trigeminal nerve reaction together with normal taste reception. The sensation of heat is caused by the food’s activating nerves that express TRPV1 and TRPA1 receptors. EVOO and Brassicaceae compounds that cause this sensation are secoiridoids from olive and allyl isothiocyanate from Brassicaceae.

## 2. Methodology

The selection of articles was conducted on the platforms of scientific research Science Direct, Scopus, PsycINFO, PubMed, and Google Scholar. Several combinations of the following terms were used: (“bitter,” “liking,” “sensory perception,” “sensory properties,” “acceptability,” “avoidance,” “bitterness,” AND (“food” OR “consumer”)) within the fields “article title, abstract and keywords”. Furthermore, cross-referencing was performed, in order to detect every article that could be instrumental for the research. The selection of the articles to be reviewed followed this pattern: After the database research and cross-referencing, a total of 1667 articles were selected. Then, a first selection, aimed at checking for duplicates, deleted 50 articles. Subsequently, the selected articles were screened according to the following conditions:

- Written in English;
- Published in peer-reviewed journals;
- Published between 2000 and 2019;
- Dealt with perception and/or preference toward bitterness in food;
- Not focused on experts’ ratings or on targeted group of consumers (such as children or elderly);
- Not aimed at evaluating new product development;
- Not aimed at only explaining the physiology of bitterness perception in consumers from a clinical point of view.

A total of 223 articles were considered for full reading. Subsequently, deep reading allowed articles to be matched with the abovementioned conditions and a final set of 99 articles were selected as suitable for the literature review. The final set of articles was divided in three sections, according to the core investigated topic:

- Psychological Studies (*n* = 19);
- Studies on basic tastes (*n* = 20);
- Studies on a single product acceptability (*n* = 60); within this section, two sub-sections were found with articles that dealt specifically with extra-virgin olive oil (EVOO) (*n* = 20) or vegetables (*n* = 12).

**Figure 2:**
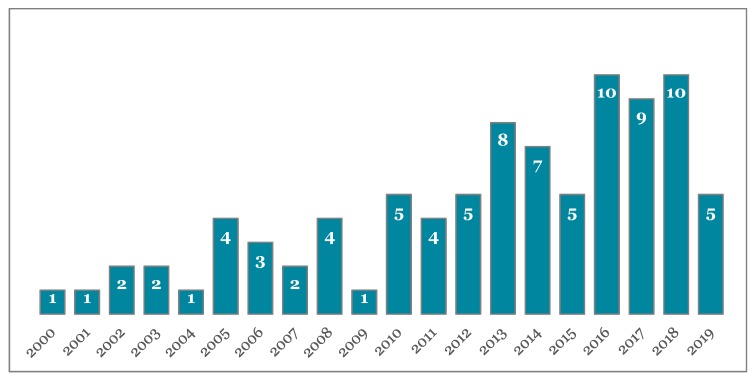
Numbers of articles per year (2000–2019).

Figure 2 depicts the temporal distribution of the reviewed articles. Although the total number of articles was quite exiguous, there was an increasing trend of papers published in the latest years. This testifies the growing interest of the scientific community toward the topic under investigation in the current review. Nevertheless, the limited number of articles shows the need for further research on specific issues that will hereby be presented.

## 3. Psychological Studies

The strong debate around bitterness also interested the psychological domain. In detail, several academic studies have investigated what the psychological reasons behind taste preference could be. Indeed, expectations and motivational states can substantially influence the taste experience of consumers, providing further explanation to the analysis of products preference and consumption [55].

A first group of researchers devoted their attention to the psychological determinants capable to affect food perception. A study by Robino et al. [56] investigated the correlation between the occurrence of alexithymia in low tasters. Alexithymia is the trait which identifies the inability of an individual to identify feelings and it is supposed to be based on the same neural mechanisms associated to food perception. Actually, non-taster individuals appeared to have higher alexithymia scores compared to other individuals. Additionally, in Garcia-Burgos and Zamora [57], the role of sensitivity of individuals is overshadowed by elements such as affective reactions and incentive properties of food. In particular, products with a psychoactive effect (such as coffee and chocolate) are preferred, regardless of their bitterness, especially in the presence of some motivational state (hunger, stress, and weight/health concern). While, it has been underlined that mental or physical stress is able to lower tastes perception [58], in detail, human taste thresholds for sour and bitter can be altered in the case of acute stress [59,60]. Besides, the presence of depressive disorder can have an enhanced disliking for bitter foods [61].

Furthermore, other studies profiled the traits characterizing consumers who tend to appreciate bitterness in food. For instance, a higher preference for bitter-tasting foods has been found in consumers who score high in openness to new foods [62]. The supposed reason for this correlation is found in the evolutionary feature of the disgust to bitterness; so adventurous consumers, who tend to detach from traditional preferences, also tend to prefer bitterness in food [63]. The consumer is primarily influenced by the neophobia towards novel foods, this trait appears at an early age and, with the years, the individual can overcome it to different degrees [64]. The reason why is represented by the potential risk recognized in foods never tried before. So, how individuals cope with innate neophobia depends upon emotional factors and a priori personal beliefs [65]. A study which analyzed how personal traits influence tastes preferences and choice found that bitterness is strictly linked with the presence of neophobia trait in consumers [60,66,67]. This has also been confirmed in Appleton et al., [68] on adolescents; this group is more likely to be neophobic compared to other consumer groups. This study also found that females, on average, appear to be more prone to eat bitter vegetables, such as the ones from Brassicaceae family. 

From a purely psychological point of view, the preference for bitter foods has been correlated with malevolent personality traits of the individuals. Specifically, Sagioglou and Greitemeyer [69] found that, among the basic taste preferences, bitter preference is the most predictive of personality traits; and the ones most associated with this preference appear to be sadism and psychopathy. Analogously, the ingestion of bitter-tasting food is supposed to foster hostile behavior in consumers, whether or not previously provoked [70].

Analyzing the way in which consumers express themselves when faced with a sensory experience in which bitterness is present, a linguistic trick has been highlighted by Lesschaeve and Noble [71]. Authors found that consumers used “bitter” as a descriptor of red wine when expressing dislike (even attributable to other negative sensory features, such as astringency or acidity), while respondents used euphemisms like “robust” or “strong” when expressing liking.

Another category of psychological studies is represented by the ones devoted to synesthesia. In fact, when several sensory stimuli are present in the environment, they are evaluated by the consumer in a heuristic and integrated way. Thus, the individuality of consumers can highly affect how they are perceived [55]. Studies belonging to this category, suggest that a soundtrack can alter consumers’ perception of bitterness in beer and cider [72,73]. While the shape of the mug, being short and narrow, is believed to enhance the bitterness perception in coffee by most consumers [74], the shape of food packaging is believed to influence expectations on taste in general, but no effect was found for the bitter taste, in particular [75]. Similarly, it has been proved that distraction in the environment (e.g., music) can alter bitterness perception, lowering it in favor of sweetness perception [76].

## 4. Basic Tastes

Taste perceptions and taste preferences have been investigated with different purposes, but mainly to explain food choices and consumption frequencies among individuals. Bitter taste is paired with danger, so the humans learnt through centuries to dislike this taste. Besides, the food industry gave further impulse to this selection of tastes through an increasing de-bittering of food, in order to please the consumer, that, in turn, reduced exposure to the bitter taste and aided in consumers further disliking it [77]. This concept can be extended to taste in general: Healthier foods are increasingly accepted by consumers, but only provided that their taste is considered pleasant [78]. 

However, this evolutionary tendency is present with a certain heterogeneity among individuals. Indeed, consumers can be categorized into different groups according to their bitter taste sensitivity, that is also considered a general taste sensitivity proxy. Tasting sensitivity of individuals, genetically determined, is measured on the perceived bitterness of a solution made with 6-n-propyl-thiouracil (PROP), allowing to categorize consumers in the subsequent groups: non-tasters, medium tasters, and super-tasters. This difference can have influence on the diet of the individual [79]. In general, high sensitivity is more likely to be found in women [80,81] and in early age [82], as there is a widespread decrease in sensitivity for bitterness with ageing [83]. While, according to Pasquet et al. [84], the different sensitivity groups have no relevant differences in preferences and disliking of foods. Indeed, the effect of the taster status has already been linked to the consumption of Brassicaceae, only for children, and no effect was detected [82]. A study by Andreeva et al. [85] devoted to the characteristics of consumers correlated with enhanced bitter food consumption, revealed that people with lower body mass index (BMI) and alcoholic beverage drinkers were the ones who consumed the most amount of bitter foods. The authors suppose that this effect can be mediated by the taster’s status. Other differences among genders can be in the link highlighted by Amman et al. [86], who found that, only in men, supertasters tended also to have a high food disgust sensitivity. 

Some further links between the sensitivity to tastes of the individuals and BMI or diet have been found. Specifically, some studies were directed at profiling the taste sensitivity of individuals belonging to different BMI categories. In detail, they found that individuals with a higher BMI are more likely to be super-tasters and, thus, sensitivity to tastes can influence diet and also health as a result [87,88]. In fact, with vegetables less sweet and more bitter, super-tasters tend to have diets lower in vegetables, and; thus, lower in healthy substances content [89]. Nevertheless, there is no wide consensus on this direct link, as in Beckett, et al. [90] this connection seems to be rejected in favor of social and cultural influences. Additionally, in Bajec and Pickering [91], no association was found between the two elements and the authors supposed that the reason lay in the absence of dietary restraints in their consumer sample. These scholars also suggest that an enhanced intake of vegetables by non-tasters can be driven by the texture of foods, over taste.

Another source of variance in consumers’ taste preferences can be represented by individuals’ general habits, traits, and demographics. In this context, a study performed by Hemmerling and Spiller [92], through a cluster analysis, has identified a group of consumers liking bitter above other tastes being, on average, adult and regular organic food buyers.

Despite a genetic variation in humans, there are also effects played by exposure on Western consumers’ tastes, as confirmed by Sorokowska et al. [93], who compared tastes preferences among different societies, and found no aversion toward the bitter taste by traditional societies from Africa and South America. Suggesting that Western consumers lost contact with this sensory property and, subsequently, increased their aversion to it.

Several studies have disclosed the relation between specific foods and each basic taste. For example, a study by Cornelis et al. [94] investigated the most bitter tasting foods, being grapefruit and grapefruit juice, liquor, beer, mustard, coffee, kale, chard, red wine, Brussels sprouts, and lemonade. Underlining that vegetables from the Brassicaceae family are recognized as extremely bitter-tasting by consumers [95]; and that mislabeling of sour or astringent foods as bitter can commonly occur in consumers [96]. Moreover, the disliking toward bitterness can lower the intake of such foods [97]. 

## 5. Single Product Perception and Preference

Since food choices and consumption motives can be different, this section reveals how bitter taste is considered at the narrow level of the single food product.

The first salient element is that, in several food products, the goods perceived as more bitter by consumers are also the ones who contain chemical substances that appear to be particularly healthy [71]. This is the case of: EVOO [5], cruciferous vegetables [85], saffron [98], whole wheat bread [99], and tea [100]. This allows one to state that, from a healthiness point of view, the presence of bitterness in food is a scientifically-proven desirable element, except for, of course, non-edible plants.

This contrasts with the fact that, generally, bitterness is a driver of disliking, as previously described. This concept has been reinforced by studies aimed at evaluating the sensory profiles of single food products [101,102,103,104,105]. For example, in [99] whole bread has been described as more bitter compared to white bread, and white bread was preferred only by medium- and super-tasters. Furthermore, Lee and Lee [106] categorized consumers in clusters according to their preferences for rice wine: All three clusters showed aversion toward bitter taste. Nevertheless, some elements that are able to lower this tendency have been found in exposure to bitterness in food [36,106,107] and related to the information about the quality characteristics of bitter foods [100]. While no effect has been found regarding the level of familiarity of the consumer with the investigated product [108].

Some exceptions leading to general liking are represented by a peculiar group of foods; the ones that have psychoactive effects. This is the case of foods as chocolate (often used as a reward), coffee (used to stay awake and active), and alcoholic beverages (often used to relax or to have fun). In detail, those effects can mediate the acceptance of the bitter taste and unfamiliar food [109,110,111,112,113,114,115,116,117]. 

While, other exceptions to general disliking of bitter food can rely on the characteristics and traits of consumers. Actually, through segmentation analysis it has been demonstrated that, in the case of instant coffee, consumers that are more likely to buy higher priced products are considered as being “coffee lovers” and appear to be more likely to be lovers of the bitter taste of coffee [110]. In Harwood et al. [111,118] it has been found that a proxy for the taster status can be consumers’ most liked chocolate (milk vs. dark); that is, consumers who liked dark chocolate were more tolerant to bitterness in food. 

We will discuss in detail the case of two vegetable foods widely diffused in the Mediterranean food culture—EVOO and vegetables from the Brassicaceae family—both characterized by specific phytochemicals having strong healthy properties and bitter-pungent taste. These two products are considered by consumers as healthy, and they are also considered as pillars of Mediterranean diet [119,120,121]. Therefore, they represent two effective case studies needing further attention by researchers. Indeed, while these products have been investigated deeply and there are wide evidences about reactions and preferences of consumers toward their taste [122], specific strategies supporting a development of their taste in line with healthy characteristics are still lacking [123]. 

### 5.1. Extra-Virgin Olive Oil (EVOO) 

**Figure 3:**
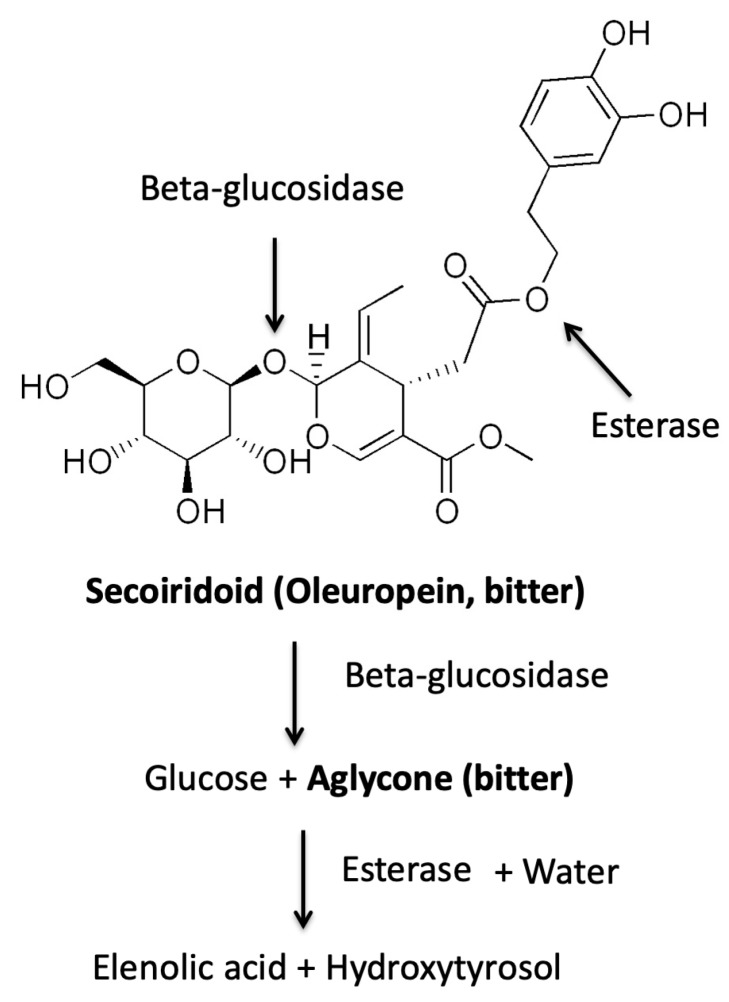
Schematic mechanism of enzyme actions (beta-glucosidase, esterase) on olive bitter secoiridoids (i.e., oleuropein). In bold chemical substances with a sensory characteristic.

The main biophenolic compounds of olive (*Olea europaea*) drupe and virgin olive oil are specific of the family of Oleaceae and related to the class of secoiridoid glycosides (oleuropein, ligstroside). When fruit tissues are damaged, beta-glycosidase endogenous enzyme activity releases from these glycosides the glucose moiety and bitter-pungent aglycones [5]. These bitter compounds can be furtherly hydrolyzed by esterase (Figure 3). 

The esterase action may occur during olive ripening, oil extraction, or oil storage without filtration, releasing phenyl alcohols (tyrosol, hydroxytyrosol) and elenolic acid from secoiridoid aglycones, with a natural loss of oil bitterness [5]. If this last hydrolytic reaction occurs in olives during over-ripening or post-harvesting storage, a very low amount of phenolic compounds will be partitioned into the oil phase during extraction, being phenyl-alcohols tyrosol and hydroxy-tyrosol, characterized by a partition coefficient favorable to the water phase [124], and then lost in olive-mill waste water. In contrast, if unripe or normal ripe olives are correctly processed, higher amounts of lipophilic aglycones are partitioned into the oily-phase ensuring a powerful antioxidant content to virgin olive oil and corresponding bitterness intensity [5]. Aglycones hydrolysis also occurs during cooking of virgin olive oil in the presence of water, as verified in different food systems (tomato sauce, canned tuna, etc.), with a consequent reduction of the bitter taste intensity in cooked food and, at the same time, preserving antioxidant and hypotensive actions of these phytochemicals [125,126].

European Regulation EU 1227/2016 normed the possibility of using sensory claims on labels, that is, taste being a possible indicator of the health content in EVOO [127]. Supporting the evolution of quality EVOO (i.e., being identified by precise sensory features). Indeed, PGI and PDO products must compel, among others, to sensory conditions to be identified as such (Regulation EU 1151/2012 updated on June 24, 2016). This tendency to backup bitterness in quality products was supported by experts’ judgements, linking positive characteristics to the presence of bitterness [128]. A problem arises considering consumers’ preferences: They do not reflect at all the view endorsed by European Regulations, with a widespread preference toward EVOO with a flat and neutral taste, as appearing from a meta-analysis that reviewed the main studies on EVOO consumers’ preferences [129].

However, the sensory properties of EVOO are addressed as being the most important element driving consumers’ preferences and purchases [130,131]. In this scenario, already studies from several years ago have proven consumers’ negative response to bitterness [132]. A recent hedonic price study of the EVOO market confirmed that a higher grade assigned by experts to the sensory profile of EVOO has a negative effect on the price structure of this product, so that the demand for a better taste by consumers does not reflect the opinion of experts [133]. Furthermore, consumers’ aversion is directed toward EVOO with a strong taste, characterized also by pungency [134], besides bitterness. On the other hand, a further sensory element used by European Regulations to describe EVOO taste (i.e., Regulation (EEC) No. 2568/91 and following ones) is the “olive fruity” flavor. This flavor is, indeed, positively perceived both by consumers [135] and by experts [133].

However, in the case of preferences toward bitterness in EVOO, exposure plays a pivotal role: In fact, the aversion toward bitter taste seems to be strictly linked with consumers’ familiarity and knowledge about the product [120,128]. Similar findings are also revealed for expert tasters [34,136]. 

Indeed, exceptions to the general aversion toward bitterness have been found in consumers with high familiarity with bitter EVOO, mainly in traditional producing and consuming areas, such as Italy and Tunisia [129,137]. This concept is reinforced by studies that analyzed consumers’ preferences for EVOO in non-traditional producing countries. For example, Japanese consumers showed low preference for products with a sensory profile characterized by bitterness [138], and the same was found in US by Delgado, Gómez-Rico, and Guinard [34]. While, in Argentina, different traditions in EVOO production were addressed as the reason for heterogeneity of preferences between the cities of Buenos Aires and Mendoza [139]. Furthermore, Recchia, Monteleone, and Tuorila [35] found that in a non-traditional EVOO producing country, such as Finland, even consumers with a strong commitment to the product are reluctant to accept a sensory profile characterized by bitterness. 

Considering the complete sensory profile of EVOO in a broader way, other elements that are believed to influence preferences toward the taste of EVOOs are feature-linked to the basic taste preferences present in consumers from birth [48,140,141,142], or learned through life with education or culture [143]. While, among the non-intrinsic characteristics of the product, brand is the one that most powerfully shapes the perceptions of other attributes [129,141]. A peculiar result has been obtained in Santosa et al. [144], where consumers from US, unfamiliar with olive oil as a product, evaluated EVOO with the same attributes of wine, in the end, they showed to be not capable enough of detecting taste differences from one product to another.

Within this context, information can also play an important role. A study performed in Italy [126] has demonstrated that when the nutritional benefits of bitter compounds of EVOOs are shown to consumers, the preferences toward bitter EVOOs change very rapidly. The reasons for the effect of health information on consumers’ preferences can be linked to the tendency of consumers to accept a less pleasant taste in food when health goals are made salient [145].

### 5.2. Vegetables from the Brassicaceae Family

**Figure 4:**
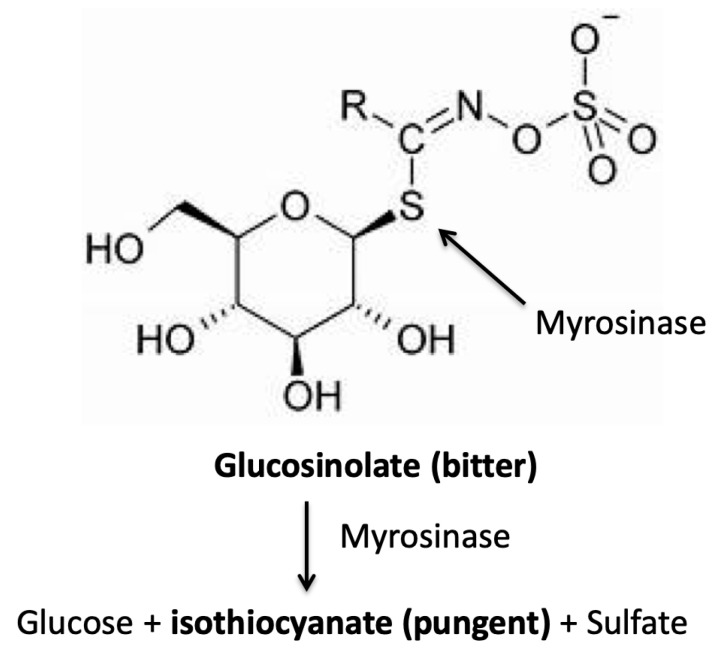
Simplified mechanism of myrosinase enzyme action in Brassicaceae on bitter glucosinolates with release of pungent isothiocyanate. In bold chemical substances with a sensory characteristic.

Bitterness is a salient characteristic of the Brassicaceae family [68,94,95]. The main bitter and pungent compounds of Brassicaceae are typical sulfur-containing phytochemicals like glucosinolates present in cell vacuoles and their hydrolysis compounds (isothiocyanates, thiocyanates, and nitriles) formed by the action of the enzyme myrosinase (or thioglucoside glycopyrrolate) when plant tissue is damaged and the cytoplasmatic enzyme can be in contact with glucosinolates [146] (Figure 4). 

The hydrolysis products have many different biological activities (e.g., as plant defense compounds and attractants). For humans, these compounds function as cancer-preventing agents and flavor compounds. The effects of cooking and thermal processing on their content and influence on sensory and nutritional properties of these vegetable products (broccoli, Brussel sprouts, cauliflower, cabbages) have been reviewed [17]. In this work, it was observed that steaming, in contrast to boiling, of Brassica vegetables ensures better preservation of glucosinolates, which are also influenced by the cooking times. In general, raw/frozen use and the way of cooking can largely influence the healthy substances content in Brassica vegetables [17,147] and also the final taste of the product. 

A study on consumers’ preferences on broccoli-based food, found that preferences of consumers can be strongly affected by the familiarity with the specific product and with bitter food in general [36]. This appears to be true for all vegetables in general, according to a recent meta-analysis [148]. However, peculiar is the case of rocket within the context of Brassicaceae, as its characteristic hot taste leads to different preferences of consumers. In fact, a study proved that preference for rocket is not dependent on the taster status of the individuals [149], the reason why lies in the preferences toward hot taste that follow a different pattern compared to bitter taste [60]. In general, we found that physical factors and individual traits, such as habits and ethnicity [95,150], lead to very different preferences among consumers. Nevertheless, preference for these vegetables is the most powerful predictor of intake [151,152]. 

At this point, the study of these products appears to be essential for the current knowledge, since they are also targeted by de-bittering or strong cooking, that is responsible for a lowered health content [153], along with the diminishing tendency of consuming vegetables from wild species that tend to be more bitter [31]. The large variability in glucosinolate content in different Brassica vegetables, the cooking effect, and food habits, are, in fact, factors which complicate consumer preferences’ analysis [154] and a deeper knowledge is needed to complete the picture. 

## 6. Conclusions and Future Projections

**Figure 5:**
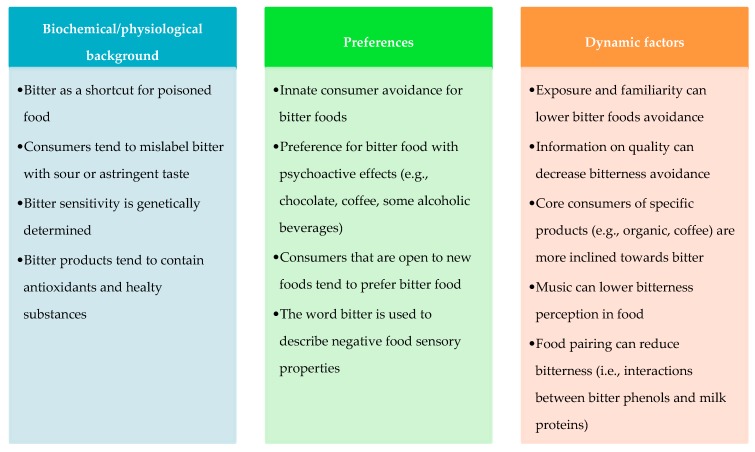
Key literature insights.

In order to have a comprehensive view about consumers’ preferences for bitter tasting vegetable foods, this review collected records from different disciplines domains, to provide a multifaceted view of the topic and to form a basis for further research developments. The collected insights, as represented in Figure 5, address three main key points raised in the review, concerning: (1) Biochemical and physiological background, (2) preferences toward bitter-tasting food, and (3) elements that have a two-way influence with preferences (dynamic factors influence preferences and that are also influenced by preferences).

The perception of bitter vegetable foods is founded on an evolutionary mechanism and; thus, it is basically avoided. The lack of exposure has led consumers to misidentify this sensory property with sour or astringent tastes. Furthermore, the perception of bitterness in food depends on individuals’ prior expectations and on their capacity of identifying tastes; this capacity, in turn, can be dependent on personality traits, such as alexithymia. The sensitivity to bitter taste (and to tastes in general) is genetically determined and can be measured with a proper laboratory solution and, sometimes, it is able to shape the preferences and thus the diet of individuals. 

Analyzing consumer preferences for bitter foods; however, we found essentially an avoidance toward all the bitter-tasting foods. Food with psychoactive effects, such as chocolate, coffee, and some alcoholic beverages, diverge, as in this case their bitterness is overwhelmed especially in the presence of particular motivational states (e.g., stress). Typically, consumers that are quite open to new foods are adventurous enough to contradict the evolutionary tendency and tend to like bitterness in food. Moreover, a malevolent personality is more likely to be found in consumers who like bitter-tasting foods. In addition, from a semiology point of view, consumers use the word “bitter” with a negative sense, while when aiming for a positive meaning they prefer to use synonyms.

Analyzing the dynamic elements that can alter the bitterness perception and preference, several elements should be underlined. 

The most important one appears to be exposure. Indeed, consumers who try bitter-tasting foods are the ones that are more likely to consume it in the future too. That is why niches of consumers that appreciate this sensory note are found among people that have bitter food consumption in their habits, tradition, or are expert tasters. While, another important driver is the information related to the products. This can be the reason why other niches are found among organic food shoppers and among “coffee lovers.” Other external elements can also influence the perception of bitterness in food, such as the shape of the coffee mug (the shorter, the more bitter) and the music in the environment acting as a distraction element and lowering bitterness perception.

Although several features characterizing the topic have been already underlined and investigated by other scholars, the present study highlights that consumer perceptions and preferences for bitter taste are still not fully identified and understood. In particular, the wealth of researches conducted on EVOO, aimed at supporting quality products, can represent a model for the future development of similar studies on vegetables such as Brassicaceae that, currently, have been quite neglected by researchers. This review, to the best of our knowledge, is the first attempt to summarize and discuss the available knowledge on Brassica vegetables.

The core limit of this review is represented by the dispersion of knowledge, which is disaggregated in several discipline domains. Thus, it would be desirable that future researches should be directed toward providing a complete picture of consumers’ behavior toward bitterness, beyond the single-product circumstances. Moreover, while extensively treated in the scientific literature, the current review does not provide an in-depth discussion about all bitter compounds in food (e.g., peptides) and their nature. 

Furthermore, we acknowledge that the present paper offers insights on both consumers’ perceptions and preferences for bitterness, which are two different concepts (as preferences are related to the purchase decision and willingness to pay and thus to the choice of a product in a shopping setting). In addition, many studies are especially focused on the hedonic evaluation of product liking; therefore, limiting their scope mostly to product acceptability. Therefore, further reviews should concentrate their efforts on the purchase decision or on the food consumption dimension. Relatedly, it would be desirable that studies concentrated on consumers’ perceptions provide detailed thresholds of perceived and accepted bitterness by consumers in food products, in order to have a clearer understanding of the levels of attribute intensity. Thus, ensuring the acceptability of foods and the ideal sensory profile preferred by the majority—or by niches—of consumers (suggesting tailored product–market strategies). 

From a practical perspective, the interaction among bitterness perception and preference, gender and age, has to be taken into account. Consumers age and gender groups represent strategic market segments characterized by different communication needs. Specific marketing/communication campaigns need to be developed to promote bitterness acceptability. Marketing strategies based on the connection between bitterness and healthiness could be successful in targeting elder consumers and women, increasing the effectiveness of communication interventions. For instance, elderly consumers have specific needs linked to their awareness of the importance of preventing non-communicable diseases. While, women, who are more likely to be super-tasters, and more prone to consume vegetables with less appealing sensory properties, could overcome their aversion with specific messages linked to health. In this context, young consumers represent the most difficult market segment to target, due to their low health interest and their stronger rejection to bitter tastes. Therefore, tailored communication strategies should be characterized by a long-run approach and should focus on food consumption habits starting from very early age, especially in school settings, in order to leverage the effect of social norms played by their peers [155,156,157]. 

In addition, an economic view of consumers’ preferences for bitter food products is completely lacking in current literature. Accordingly, future studies could include the estimation of monetary values assigned by consumers to bitter sensory properties (and exploring the relations of these values with individual characteristics), providing valuable information for scholars and practitioners. 

Moreover, the health benefits of bitter foods, underlined by some of the studies included in this review, reveal the need for specific strategies aimed at valorizing the key role of these products in a healthier diet. Indeed, these strategies represent an important opportunity, as bitterness perception and preference insights can be used to develop specific campaigns aimed at promoting bitter food consumption to improve populations’ well-being.

Among all the possible products that could be targeted by promotional campaigns, we recognized Brassica vegetables and EVOO as needing particular attention. In fact, the breeding and food industry are driving their taste evolution towards the majority of consumers’ preferences reducing their health content, causing both a negative impact on the society as a whole and enhancing the gap between the value perceived by producers and consumers on “high sensory and nutritional quality products”. Whereas, effectively communicating the positive relation between bitter and health benefits could be a straightforward strategy to increase the intake and valorization of these products. 

A last consideration should be made about the role of a deeper knowledge in consumers for the traditional and local uses of vegetables, recipes, cooking methods, and food pairing (Brassicaceae and EVOO are not consumed as whole single products but normally in combination with other ingredients in different dishes). The bitter sensory perception of these whole or cooked bitter ingredients, in fact, due to the chemical interactions of bitter molecules with other ingredients, can be dramatically modified by cooking and/or mixing with ingredients able to form chemical complexes with bitter molecules with an evident masking effect on our tongue [158,159]. As an example, the molecular understanding on EVOO bitter compounds interactions with other food ingredients (e.g., milk proteins in dairy products, tomato, etc.) [160] should contribute to increase the acceptability of healthy, bitter VOOs and vegetables. The molecular interactions between EVOO bitter biophenols and dairy ingredients, as well as the preparation/cooking systems, in fact, can contribute to increase sensory harmony of dishes by reducing the bitter taste without altering the nutritional properties and bioavailability of bitter phytochemicals [125].
